# Crystal structure of chlorido­[diphen­yl(thio­phen-2-yl)phosphine-κ*P*]gold(I)

**DOI:** 10.1107/S2056989022009227

**Published:** 2022-09-26

**Authors:** Thomas Neils, Andrew LaDuca, John E. Bender, Richard J. Staples, Shannon M. Biros

**Affiliations:** a Department of Chemistry, Grand Valley State University, Allendale, MI 49401, USA; b Center for Crystallographic Research, Department of Chemistry, Michigan State University, East Lansing, MI 48824, USA; Purdue University, USA

**Keywords:** crystal structure, gold complex, triaryl phosphine ligand, C—H⋯π inter­action, disorder

## Abstract

The crystal structure of the title compound, [AuCl(C_16_H_13_PS)], is reported. The mol­ecular structure features a nearly linear arrangement of the chloride and phosphino ligands around the gold(I) center, with a P—Au—Cl bond angle of 179.42 (9)°. The Au—P and Au—Cl bond lengths are 2.226 (2) and 2.287 (2) Å, respectively. The geometry of the groups bonded to the phospho­rus atom of the ligand is a slightly distorted tetra­hedron. The phenyl and thienyl rings of the ligand are extensively disordered, with the thienyl refined over all three possible positions on the phospho­rus atom. The relative occupancy ratio between these positions was found to be 0.406 (3):0.406 (2):0.188 (2). One of the major thienyl ring positions with the relative occupancy of 0.406 was modeled as two rotational isomers around the C—P bond with a relative occupancy ratio of 0.278 (3):0.128 (3). Inter­molecular C—H⋯π inter­actions present in the crystal lattice link mol­ecules of the title compound together to form a complex three-dimensional network.

## Abstract 2

The crystal structure of the title gold(I) complex is described. The geometry around the gold atom is nearly linear, and the phospho­rus atom of the ligand adopts a slightly distorted tetra­hedral geometry. The structure features extensive disorder of the thienyl and phenyl groups with the thienyl substituent disordered over all three possible positions. Mol­ecules of the title compound are held together in the solid state *via* a variety of inter­molecular C—H⋯π inter­actions.

## 1. Chemical context

The incorporation of tri­aryl­phosphines as ligands in metal complexes has led to a multitude of species capable of, for example, catalyzing organic transformations, binding to biological targets, and combating cancer. The synthesis of unsymmetric tri­aryl­phosphines has the potential to add additional functionality and selectivity to the resultant metal–ligand complexes. If we consider gold(I)–PAr_3_ complexes, structural diversity of the phosphine ligand has led to properties such as selective catalysis for cyclo­isomerization reactions (Christian *et al.*, 2017 ▸), triboluminescence (Kuchison *et al.*, 2009 ▸), and enzyme inhibition (Zhang *et al.*, 2014 ▸; Fonteh & Meyer, 2009 ▸). To this end, our group has been developing synthetic routes to unsymmetric tri­aryl­phosphines, their chalcogenide derivatives and the resultant metal–ligand complexes (Luster *et al.*, 2022 ▸). While attempting to prepare a complex between gold(I) and the selenide derivative of diphenyl-2-thienylphosphine, we isolated single crystals of the title compound as a decomposition product. 
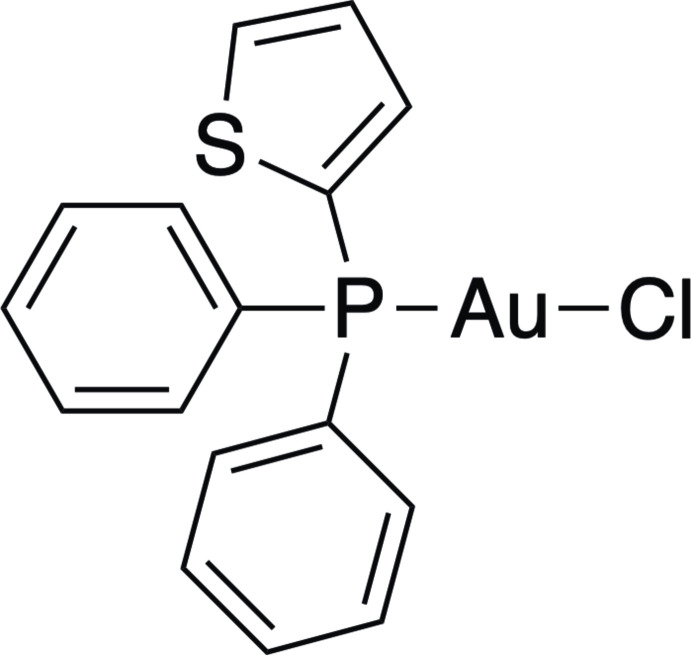


## 2. Structural commentary

**Figure 1:**
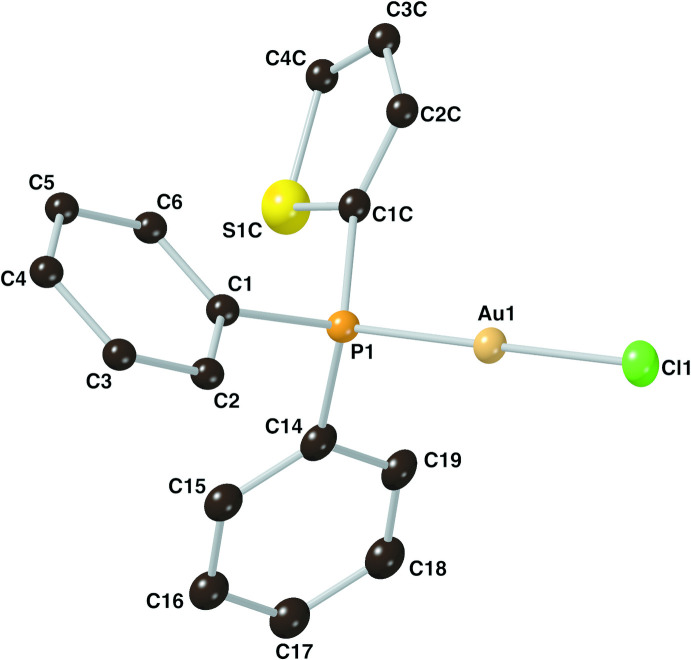
The mol­ecular structure of the title compound **I**, with the atom-labeling scheme. Displacement ellipsoids are drawn at the 30% probability level, all hydrogen atoms have been omitted and only the predominant Part 1 is shown for clarity.

The structure of compound **I** was solved in the ortho­rhom­bic space group *P*2_1_2_1_2_1_ with a Flack parameter of −0.002 (6) (Parsons *et al.*, 2013 ▸). The phospho­rus–gold and chloride–gold bond lengths are 2.226 (2) and 2.287 (2) Å, respectively. The phenyl and thienyl rings of the ligand are disordered, with the thienyl ring being distributed over all three possible positions at the P atom. The relative occupancy ratio between these positions was found to be 0.406 (3):0.406 (2):0.188 (2). Furthermore, the thienyl ring position with a relative occupancy of 0.406 (3) was modeled as two rotational isomers around the C—P bond with a relative occupancy ratio of 0.279 (3):0.128 (3) (see the *Refinement* section for further details of the treatment of the disorder). The atom-labeling scheme for the predominant moiety (Part 1: phenyl rings C1–C6 and C14–C19 as well as thienyl ring S1*C* and C1*C*–C4*C*) is shown in Fig. 1 ▸.

The coordination geometry of the gold center is nearly linear with a P1—Au1—Cl1 bond angle of 179.42 (9)°. With regard to the phosphine ligand, for the most prevalent moiety the P—C bond lengths are 1.769 (7), 1.786 (7) and 1.874 (14) Å. The geometry around the phospho­rus atom P1 resembles a tetra­hedron with a τ_4_ descriptor for fourfold coordination of 0.95 (where 0.00 = square planar, 0.85 = trigonal pyramidal, and 1.00 = tetra­hedral; Yang *et al.*, 2007 ▸). The C—P—C bond angles range from 105.3 (6) to 106.9 (11)°, and the Au—P—C bond angles range from 111.9 (5) to 113.6 (3)° for the most prelavent moiety.

## 3. Supra­molecular features

**Figure 2:**
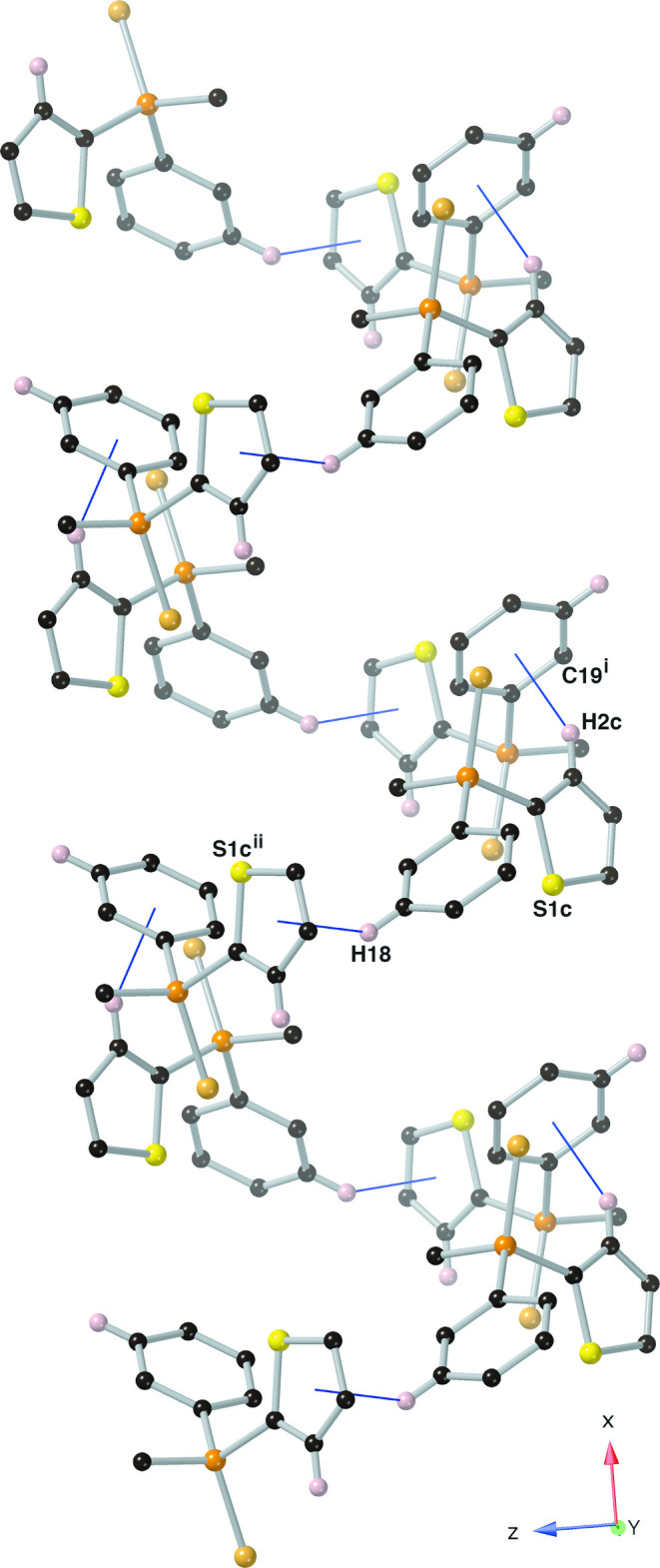
The C—H⋯π inter­actions (solid, blue lines) found in crystals of the title compound that form helices that run along the *a*-axis direction, depicted using a ball-and-stick model with standard CPK colors (Au = tan , hydrogen = light pink). The chlorine atoms, phenyl ring C1–C6, and any hydrogen atom not involved in a C—H⋯π inter­action have been omitted for clarity. Only Part 1 is shown. Symmetry codes as in Table 1.

**Figure 3:**
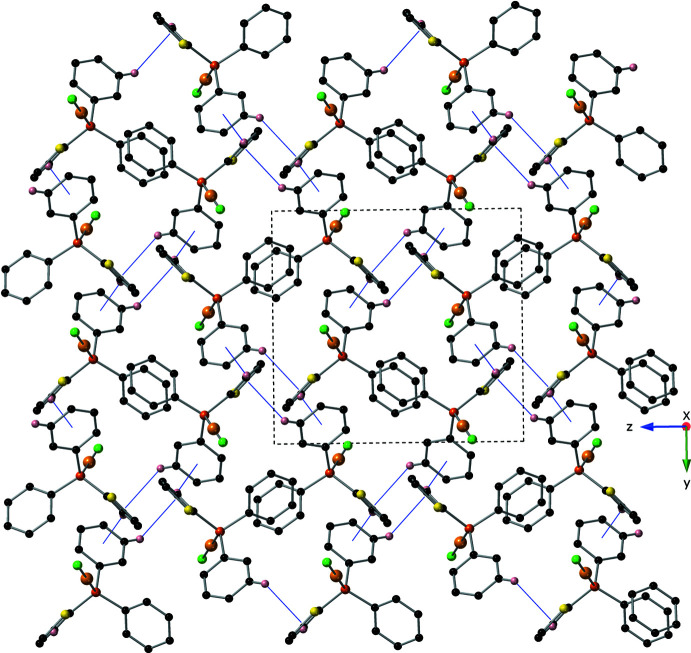
The crystal packing of the title compound as viewed down the *a*-axis, depicted using a ball-and-stick model with standard CPK colors (Au = tan , Cl = green, H = light pink). Inter­molecular C—H⋯π inter­actions are shown with solid, blue lines. For clarity any hydrogen atoms not involved in a C—H⋯π inter­action have been omitted. Only Part 1 is shown.

Individual mol­ecules of the title compound are held together through inter­molecular C—H⋯π inter­actions (Table 1 ▸). In Part 1, these inter­actions exist between the C14–C19 phenyl ring and the hydrogen atom C2*C*(H2*C*) of the thienyl ring as well as between hydrogen atom C18(H18) and the S1*C*/C1*C*–C4*C* thienyl ring. These inter­molecular C—H⋯π inter­actions link the mol­ecules together to form helices that propagate along the *a*-axis direction (Fig. 2 ▸). The helices are then held together through C—H⋯π inter­actions to form a complex 3D network (Fig. 3 ▸). The remainder of the inter­molecular C—H⋯π inter­actions present in this structure are not exclusive to Part 1, and are listed in Table 1 ▸.

## 4. Database survey

A search of the Cambridge Structural Database (CSD, Version 5.42, November, 2020; Groom *et al.*, 2016 ▸) for structures containing a P—Au bond where the phospho­rus atom bears one thienyl ring resulted in 14 hits. Structures IHUJUQ (Ho & Tiekink, 2003 ▸) and IHUJUQ01 (Monkowius *et al.*, 2003 ▸) are closely related to compound **I**, with a linear arrangement of chloride and one tris­(2-thien­yl)-substituted phosphine ligand bound to a gold(I) atom. Another related structure is IWAYUC (Yang *et al.*, 2016 ▸), which contains a di­phenyl­phosphino-3-thienyl-1*H*-imidazole ligand again bound to a gold(I) atom that also bears a chloride. Finally, structure YAHPUT (Stott *et al.*, 2005 ▸) features a terthio­phene-substituted di­phenyl­phosphinogold(I)–chloride complex.

## 5. Synthesis and crystallization

A small vial was charged with diphen­yl(2-thien­yl)phosphine selenide (10-15 mg; Luster *et al.*, 2022 ▸) and a stoichiometric amount of chloro­(tetra­hydro­thio­phene)­gold(I). The solids were dissolved in 1 mL of CDCl_3_, and the reaction mixture was transferred to an NMR tube. Crystals of compound **I** were grown serendipitously *via* slow evaporation of the solvent.

## 6. Refinement

Crystal data, data collection and structure refinement details are summarized in Table 2 ▸. All hydrogen atoms were placed in calculated positions and refined as riding: C—H = 0.95–1.00 Å with *U*_iso_(H) = 1.2*U*_eq_(C). The electron density corresponding to the disordered phenyl rings and the thienyl ring was modeled over three parts. In the model, electron density corresponding to the thienyl ring was found at three positions on the phospho­rus atom. In one of these positions, the thienyl ring was also found to be present as two rotational isomers corresponding to a 180° rotation around the C—P bond. The relative occupancies of each position of the thienyl ring were refined, while the total occupancy of all thienyl sites as well as the occupancy sum of each site were constrained to unity using SUMP commands. The thienyl occupancy rates refined to be 0.406 (2):0.278 (3):0.128 (3):0.188 (2) for the sites of S1*C*, S1*B*, S1*A* and S1*D*. Bond lengths and angles of all four thienyl moieties were restrained to be similar to each other using *SHELXL* (Sheldrick, 2015*b* ▸) SAME commands with an esd of 0.001 Å. For the pivot moiety with the highest occupancy (S1*C/*C1*C*–C4*C*), distance restraints were used to ensure a model with bond lengths and angles that agree with known values. Bonds of the thienyl ring were restrained using DFIX commands to be 1.70 (S1*C*—C1*C*), 1.34 (C1*C*—C2*C*, C3*C*—C4*C*) and 1.41 (C2*C*—C3*C*) Å with an esd of 0.002 Å in *SHELXL* (Sheldrick, 2015*b* ▸). The less occupied thienyl rings *A* and *B* were also restrained to be planar and coplanar with the P atom using FLAT commands. All P1—C_*ipso*_ distances were restrained to be similar to each other using SADI commands. The atoms of each phenyl ring C1–C6, C7–C13 and C14–C15 were constrained to resemble an ideal hexa­gon with C—C bond lengths of 1.39 Å using *SHELXL* AFIX 66 commands. Lastly, *U* ^ij^ components of all C, S and P atoms were restrained to be similar to each other for atoms closer than 2.0 Å with an esd of 0.002 Å^2^.

## Supplementary Material

Crystal structure: contains datablock(s) I. DOI: 10.1107/S2056989022009227/zl5036sup1.cif

Structure factors: contains datablock(s) I. DOI: 10.1107/S2056989022009227/zl5036Isup2.hkl

CCDC reference: 1848959

Additional supporting information:  crystallographic information; 3D view; checkCIF report

## Figures and Tables

**Table 1:** C—H⋯π interactions (Å, °) *Cg*1, *Cg*2, *Cg*3, and *Cg*4 are the centroids of the S1*C*/C1*C*–C4*C*, S1*D*/C1*D*–C4*D*, C7–C12, and C14–C19 rings, respectively.

*D*—H⋯*A*	*D*—H	H⋯*A*	*D*⋯*A*	*D*—H⋯*A*
C2*C*—H2*C*⋯*Cg*2^i^	0.95	2.80	141	4 (1)
C2*C*—H2*C*⋯*Cg*4^i^	0.95	2.79	139	4 (1)
C3*D*—H3*D*⋯*Cg*1^ii^	0.95	2.87	141	4 (1)
C3*D*—H3*D*⋯*Cg*3^ii^	0.95	2.85	141	4 (1)
C8—H8⋯*Cg*2^i^	0.95	2.80	131	4 (1)
C8—H8⋯*Cg*4^i^	0.95	2.77	130	4 (1)
C18—H18⋯*Cg*1^ii^	0.95	2.96	129	4 (1)
C18—H18⋯*Cg*3^ii^	0.95	2.95	128	4 (1)

Symmetry codes: (i) 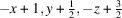 ; (ii) 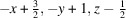 .

**Table 2:** Experimental details

Crystal data	
Chemical formula	[AuCl(C_16_H_13_PS)]
*M*_r_	500.71
Crystal system, space group	Orthorhombic, *P*2_1_2_1_2_1_
Temperature (K)	173
*a*, *b*, *c* (Å)	10.0322 (13), 12.0784 (15), 12.9412 (16)
*V* (Å^3^)	1568.1 (3)
*Z*	4
Radiation type	Mo *K*α
μ (mm^−1^)	9.77
Crystal size (mm)	0.24 × 0.16 × 0.11

Data collection	
Diffractometer	Bruker APEXII CCD
Absorption correction	Multi-scan (*SADABS*; Krause *et al.*, 2015 ▸)
*T*_min_, *T*_max_	0.474, 0.745
No. of measured, independent and observed [*I* > 2σ(*I*)] reflections	13335, 3075, 2854
*R*_int_	0.037
(sin θ/λ)_max_ (Å^−1^)	0.617

Refinement	
*R*[*F* ^2^ > 2σ(*F* ^2^)], *wR*(*F* ^2^), *S*	0.025, 0.059, 1.08
No. of reflections	3075
No. of parameters	341
No. of restraints	837
H-atom treatment	H-atom parameters constrained
Δρ_max_, Δρ_min_ (e Å^−3^)	1.02, −0.53
Absolute structure	Flack *x* determined using 1149 quotients [(*I* ^+^)−(*I* ^−^)]/[(*I* ^+^)+(*I* ^−^)] (Parsons *et al.*, 2013 ▸)
Absolute structure parameter	−0.002 (6)

Computer programs: *APEX2* and *SAINT* (Bruker, 2013 ▸), *SHELXT2018/2* (Sheldrick, 2015*a* ▸), *SHELXL2019/2* (Sheldrick, 2015*b* ▸), *CrystalMaker* (Palmer, 2007 ▸), and *OLEX2* (Dolomanov *et al.*, 2009 ▸; Bourhis *et al.*, 2015 ▸).

## supplementary crystallographic information

### Crystal data

**Table:**

[AuCl(C_16_H_13_PS)]	*D*_x_ = 2.121 Mg m^−^^3^
*M**_r_* = 500.71	Mo *K*α radiation, λ = 0.71073 Å
Orthorhombic, *P*2_1_2_1_2_1_	Cell parameters from 8845 reflections
*a* = 10.0322 (13) Å	θ = 2.3–26.0°
*b* = 12.0784 (15) Å	µ = 9.77 mm^−^^1^
*c* = 12.9412 (16) Å	*T* = 173 K
*V* = 1568.1 (3) Å^3^	Block, clear colourless
*Z* = 4	0.24 × 0.16 × 0.11 mm
*F*(000) = 944	

### Data collection

**Table:**

Bruker APEXII CCD diffractometer	2854 reflections with *I* > 2σ(*I*)
φ and ω scans	*R*_int_ = 0.037
Absorption correction: multi-scan (SADABS; Krause *et al.*, 2015)	θ_max_ = 26.0°, θ_min_ = 2.3°
*T*_min_ = 0.474, *T*_max_ = 0.745	*h* = −12→12
13335 measured reflections	*k* = −14→14
3075 independent reflections	*l* = −15→15

### Refinement

**Table:**

Refinement on *F*^2^	Secondary atom site location: difference Fourier map
Least-squares matrix: full	Hydrogen site location: inferred from neighbouring sites
*R*[*F*^2^ > 2σ(*F*^2^)] = 0.025	H-atom parameters constrained
*wR*(*F*^2^) = 0.059	*w* = 1/[σ^2^(*F*_o_^2^) + (0.0234*P*)^2^ + 0.2535*P*] where *P* = (*F*_o_^2^ + 2*F*_c_^2^)/3
*S* = 1.08	(Δ/σ)_max_ = 0.001
3075 reflections	Δρ_max_ = 1.02 e Å^−^^3^
341 parameters	Δρ_min_ = −0.53 e Å^−^^3^
837 restraints	Absolute structure: Flack *x* determined using 1149 quotients [(*I*^+^)-(*I*^-^)]/[(*I*^+^)+(*I*^-^)] (Parsons *et al.*, 2013)
Primary atom site location: dual	Absolute structure parameter: −0.002 (6)

### Special details

**Table:**

Geometry. All esds (except the esd in the dihedral angle between two l.s. planes) are estimated using the full covariance matrix. The cell esds are taken into account individually in the estimation of esds in distances, angles and torsion angles; correlations between esds in cell parameters are only used when they are defined by crystal symmetry. An approximate (isotropic) treatment of cell esds is used for estimating esds involving l.s. planes.

### Fractional atomic coordinates and isotropic or equivalent isotropic displacement parameters (Å^2^)

**Table:**

	*x*	*y*	*z*	*U*_iso_*/*U*_eq_	Occ. (<1)
Au1	0.33353 (3)	0.56862 (2)	0.75765 (2)	0.03375 (11)	
Cl1	0.1218 (2)	0.5105 (2)	0.79464 (19)	0.0511 (6)	
P1	0.5388 (2)	0.62695 (16)	0.72152 (16)	0.0324 (4)	
C14	0.6564 (8)	0.5173 (6)	0.7053 (6)	0.0351 (9)	0.812 (2)
C15	0.6753 (8)	0.4457 (6)	0.7881 (4)	0.0368 (11)	0.812 (2)
H15	0.627496	0.456824	0.850642	0.044*	0.812 (2)
C16	0.7640 (7)	0.3578 (5)	0.7794 (4)	0.0375 (11)	0.812 (2)
H16	0.776914	0.308886	0.836035	0.045*	0.812 (2)
C17	0.8339 (7)	0.3415 (5)	0.6879 (5)	0.0377 (11)	0.812 (2)
H17	0.894563	0.281466	0.682016	0.045*	0.812 (2)
C18	0.8150 (8)	0.4131 (6)	0.6051 (4)	0.0371 (11)	0.812 (2)
H18	0.862795	0.401984	0.542603	0.045*	0.812 (2)
C19	0.7263 (9)	0.5010 (6)	0.6138 (5)	0.0363 (10)	0.812 (2)
H19	0.713377	0.549922	0.557208	0.044*	0.812 (2)
S1D	0.7040 (12)	0.4155 (10)	0.7816 (9)	0.0374 (10)	0.188 (2)
C1D	0.667 (4)	0.514 (2)	0.6928 (18)	0.0356 (10)	0.188 (2)
C2D	0.744 (4)	0.493 (3)	0.610 (2)	0.0362 (11)	0.188 (2)
H2D	0.746278	0.539222	0.550884	0.043*	0.188 (2)
C3D	0.821 (4)	0.396 (2)	0.6202 (18)	0.0370 (11)	0.188 (2)
H3D	0.869591	0.365043	0.564353	0.044*	0.188 (2)
C4D	0.819 (3)	0.351 (3)	0.7147 (17)	0.0375 (11)	0.188 (2)
H4D	0.873830	0.292031	0.738489	0.045*	0.188 (2)
C7	0.6082 (10)	0.7125 (13)	0.8182 (11)	0.0358 (9)	0.595 (2)
C8	0.5277 (8)	0.7756 (12)	0.8828 (10)	0.0351 (10)	0.595 (2)
H8	0.433508	0.772389	0.876081	0.042*	0.595 (2)
C9	0.5851 (9)	0.8435 (9)	0.9573 (8)	0.0363 (11)	0.595 (2)
H9	0.530123	0.886600	1.001469	0.044*	0.595 (2)
C10	0.7230 (9)	0.8482 (8)	0.9672 (7)	0.0372 (12)	0.595 (2)
H10	0.762189	0.894567	1.018065	0.045*	0.595 (2)
C11	0.8034 (8)	0.7851 (10)	0.9025 (8)	0.0380 (11)	0.595 (2)
H11	0.897640	0.788321	0.909272	0.046*	0.595 (2)
C12	0.7460 (10)	0.7172 (11)	0.8281 (9)	0.0381 (10)	0.595 (2)
H12	0.801027	0.674109	0.783882	0.046*	0.595 (2)
S1A	0.6318 (19)	0.8216 (16)	0.5903 (12)	0.0373 (9)	0.128 (3)
C1A	0.527 (3)	0.712 (2)	0.600 (2)	0.0365 (9)	0.128 (3)
C2A	0.450 (3)	0.714 (3)	0.515 (2)	0.0372 (10)	0.128 (3)
H2A	0.378806	0.663474	0.504209	0.045*	0.128 (3)
C3A	0.482 (3)	0.798 (2)	0.444 (2)	0.0374 (10)	0.128 (3)
H3A	0.438606	0.807240	0.379366	0.045*	0.128 (3)
C4A	0.581 (3)	0.864 (3)	0.4765 (19)	0.0373 (10)	0.128 (3)
H4A	0.616245	0.925573	0.439705	0.045*	0.128 (3)
S1B	0.4495 (10)	0.6766 (7)	0.5027 (7)	0.0381 (9)	0.278 (3)
C1B	0.539 (3)	0.7187 (17)	0.6068 (13)	0.0365 (9)	0.278 (3)
C2B	0.592 (3)	0.8176 (17)	0.5838 (16)	0.0370 (10)	0.278 (3)
H2B	0.639402	0.861263	0.632876	0.044*	0.278 (3)
C3B	0.572 (3)	0.8513 (18)	0.4807 (15)	0.0373 (10)	0.278 (3)
H3B	0.608977	0.916836	0.451653	0.045*	0.278 (3)
C4B	0.496 (3)	0.7797 (15)	0.4285 (15)	0.0374 (10)	0.278 (3)
H4B	0.471660	0.787607	0.357890	0.045*	0.278 (3)
C1	0.5435 (15)	0.7063 (10)	0.6066 (7)	0.0363 (9)	0.594 (3)
C2	0.4658 (12)	0.6722 (8)	0.5236 (8)	0.0376 (9)	0.594 (3)
H2	0.417715	0.604513	0.527384	0.045*	0.594 (3)
C3	0.4584 (10)	0.7370 (8)	0.4351 (7)	0.0377 (10)	0.594 (3)
H3	0.405272	0.713702	0.378334	0.045*	0.594 (3)
C4	0.5288 (11)	0.8360 (7)	0.4295 (6)	0.0371 (10)	0.594 (3)
H4	0.523707	0.880305	0.369030	0.045*	0.594 (3)
C5	0.6065 (11)	0.8701 (7)	0.5126 (7)	0.0373 (10)	0.594 (3)
H5	0.654586	0.937720	0.508777	0.045*	0.594 (3)
C6	0.6139 (14)	0.8052 (10)	0.6011 (7)	0.0372 (9)	0.594 (3)
H6	0.667031	0.828532	0.657829	0.045*	0.594 (3)
S1C	0.7765 (6)	0.7214 (6)	0.8449 (5)	0.0391 (9)	0.406 (2)
C1C	0.6089 (7)	0.716 (2)	0.8269 (19)	0.0359 (9)	0.406 (2)
C2C	0.5458 (15)	0.7872 (18)	0.8898 (16)	0.0353 (11)	0.406 (2)
H2C	0.451912	0.796796	0.892307	0.042*	0.406 (2)
C3C	0.6375 (13)	0.8457 (16)	0.9518 (14)	0.0365 (11)	0.406 (2)
H3C	0.611592	0.898620	1.002304	0.044*	0.406 (2)
C4C	0.7646 (14)	0.8193 (16)	0.9322 (14)	0.0375 (11)	0.406 (2)
H4C	0.838950	0.853177	0.964973	0.045*	0.406 (2)

### Atomic displacement parameters (Å^2^)

**Table:**

	*U*^11^	*U*^22^	*U*^33^	*U*^12^	*U*^13^	*U*^23^
Au1	0.03260 (17)	0.03393 (17)	0.03470 (17)	−0.00391 (13)	0.00094 (16)	0.00163 (15)
Cl1	0.0410 (13)	0.0613 (15)	0.0511 (14)	−0.0158 (12)	0.0081 (10)	−0.0031 (12)
P1	0.0345 (10)	0.0308 (9)	0.0319 (10)	−0.0026 (8)	−0.0018 (8)	0.0000 (8)
C14	0.0355 (18)	0.0308 (18)	0.0391 (17)	−0.0009 (17)	−0.0010 (16)	−0.0016 (16)
C15	0.037 (2)	0.032 (2)	0.0414 (19)	0.001 (2)	−0.0009 (19)	−0.0015 (19)
C16	0.037 (2)	0.033 (2)	0.043 (2)	0.001 (2)	0.000 (2)	−0.001 (2)
C17	0.037 (2)	0.033 (2)	0.043 (2)	0.000 (2)	−0.001 (2)	−0.0026 (19)
C18	0.037 (2)	0.033 (2)	0.042 (2)	0.000 (2)	−0.0001 (19)	−0.0017 (19)
C19	0.036 (2)	0.032 (2)	0.041 (2)	0.0000 (19)	−0.0010 (18)	−0.0017 (18)
S1D	0.038 (2)	0.033 (2)	0.0419 (18)	0.0001 (18)	−0.0007 (18)	−0.0017 (18)
C1D	0.0360 (19)	0.0315 (18)	0.0393 (18)	−0.0006 (17)	−0.0011 (17)	−0.0014 (17)
C2D	0.036 (2)	0.032 (2)	0.041 (2)	0.000 (2)	−0.0007 (19)	−0.0017 (19)
C3D	0.037 (2)	0.032 (2)	0.042 (2)	0.000 (2)	−0.001 (2)	−0.002 (2)
C4D	0.037 (2)	0.033 (2)	0.042 (2)	0.000 (2)	0.000 (2)	−0.002 (2)
C7	0.0374 (18)	0.0366 (17)	0.0336 (18)	−0.0053 (17)	−0.0045 (16)	0.0003 (15)
C8	0.037 (2)	0.036 (2)	0.033 (2)	−0.005 (2)	−0.0052 (19)	0.0002 (18)
C9	0.038 (2)	0.037 (2)	0.034 (2)	−0.005 (2)	−0.005 (2)	−0.0004 (18)
C10	0.039 (2)	0.039 (2)	0.034 (2)	−0.006 (2)	−0.004 (2)	−0.001 (2)
C11	0.039 (2)	0.040 (2)	0.035 (2)	−0.006 (2)	−0.005 (2)	−0.0011 (18)
C12	0.039 (2)	0.0398 (19)	0.035 (2)	−0.0060 (19)	−0.0055 (19)	−0.0005 (17)
S1A	0.0404 (18)	0.0373 (18)	0.0342 (18)	−0.0033 (17)	−0.0043 (17)	0.0025 (16)
C1A	0.0396 (17)	0.0364 (16)	0.0337 (17)	−0.0032 (16)	−0.0043 (15)	0.0022 (15)
C2A	0.0405 (18)	0.0372 (18)	0.0339 (18)	−0.0033 (17)	−0.0049 (17)	0.0024 (16)
C3A	0.0408 (19)	0.0376 (18)	0.0340 (18)	−0.0034 (17)	−0.0049 (17)	0.0026 (17)
C4A	0.0405 (19)	0.0374 (19)	0.0340 (19)	−0.0034 (18)	−0.0045 (17)	0.0025 (17)
S1B	0.0414 (18)	0.0384 (17)	0.0344 (18)	−0.0036 (16)	−0.0055 (16)	0.0027 (15)
C1B	0.0395 (17)	0.0363 (16)	0.0336 (17)	−0.0031 (16)	−0.0043 (15)	0.0022 (15)
C2B	0.0401 (19)	0.0371 (18)	0.0339 (18)	−0.0032 (17)	−0.0045 (17)	0.0025 (16)
C3B	0.0406 (19)	0.0374 (18)	0.0340 (19)	−0.0034 (18)	−0.0046 (17)	0.0026 (17)
C4B	0.0408 (19)	0.0376 (18)	0.0339 (18)	−0.0034 (17)	−0.0048 (17)	0.0026 (17)
C1	0.0393 (17)	0.0361 (16)	0.0334 (16)	−0.0029 (16)	−0.0042 (15)	0.0021 (15)
C2	0.0410 (18)	0.0377 (17)	0.0341 (18)	−0.0034 (17)	−0.0052 (16)	0.0027 (16)
C3	0.0411 (19)	0.0378 (19)	0.0341 (19)	−0.0037 (18)	−0.0050 (17)	0.0025 (17)
C4	0.040 (2)	0.037 (2)	0.034 (2)	−0.0034 (19)	−0.0045 (18)	0.0024 (18)
C5	0.0406 (19)	0.0373 (18)	0.0339 (19)	−0.0034 (18)	−0.0045 (17)	0.0027 (17)
C6	0.0403 (18)	0.0372 (18)	0.0341 (18)	−0.0034 (17)	−0.0043 (17)	0.0027 (16)
S1C	0.0392 (19)	0.0415 (17)	0.0367 (18)	−0.0068 (17)	−0.0065 (16)	−0.0008 (15)
C1C	0.0373 (18)	0.0367 (17)	0.0337 (18)	−0.0054 (17)	−0.0048 (17)	0.0002 (16)
C2C	0.037 (2)	0.036 (2)	0.033 (2)	−0.005 (2)	−0.0052 (19)	0.0000 (18)
C3C	0.038 (2)	0.038 (2)	0.034 (2)	−0.006 (2)	−0.005 (2)	−0.0006 (18)
C4C	0.039 (2)	0.039 (2)	0.035 (2)	−0.006 (2)	−0.0050 (19)	−0.0010 (18)

### Geometric parameters (Å, º)

**Table:**

Au1—Cl1	2.287 (2)	C11—C12	1.3900
Au1—P1	2.226 (2)	C12—H12	0.9500
P1—C14	1.786 (5)	S1A—C1A	1.699 (3)
P1—C1D	1.907 (18)	S1A—C4A	1.640 (15)
P1—C7	1.766 (7)	C1A—C2A	1.342 (3)
P1—C1A	1.881 (18)	C2A—H2A	0.9500
P1—C1B	1.852 (16)	C2A—C3A	1.410 (3)
P1—C1	1.769 (7)	C3A—H3A	0.9500
P1—C1C	1.874 (14)	C3A—C4A	1.339 (3)
C14—C15	1.3900	C4A—H4A	0.9500
C14—C19	1.3900	S1B—C1B	1.699 (3)
C15—H15	0.9500	S1B—C4B	1.640 (15)
C15—C16	1.3900	C1B—C2B	1.342 (3)
C16—H16	0.9500	C2B—H2B	0.9500
C16—C17	1.3900	C2B—C3B	1.410 (3)
C17—H17	0.9500	C3B—H3B	0.9500
C17—C18	1.3900	C3B—C4B	1.339 (3)
C18—H18	0.9500	C4B—H4B	0.9500
C18—C19	1.3900	C1—C2	1.3900
C19—H19	0.9500	C1—C6	1.3900
S1D—C1D	1.699 (3)	C2—H2	0.9500
S1D—C4D	1.640 (15)	C2—C3	1.3900
C1D—C2D	1.342 (3)	C3—H3	0.9500
C2D—H2D	0.9500	C3—C4	1.3900
C2D—C3D	1.410 (3)	C4—H4	0.9500
C3D—H3D	0.9500	C4—C5	1.3900
C3D—C4D	1.339 (3)	C5—H5	0.9500
C4D—H4D	0.9500	C5—C6	1.3900
C7—C8	1.3900	C6—H6	0.9500
C7—C12	1.3900	S1C—C1C	1.699 (3)
C8—H8	0.9500	S1C—C4C	1.640 (15)
C8—C9	1.3900	C1C—C2C	1.342 (3)
C9—H9	0.9500	C2C—H2C	0.9500
C9—C10	1.3900	C2C—C3C	1.410 (3)
C10—H10	0.9500	C3C—H3C	0.9500
C10—C11	1.3900	C3C—C4C	1.339 (3)
C11—H11	0.9500	C4C—H4C	0.9500
			
P1—Au1—Cl1	179.42 (9)	C7—C12—H12	120.0
C14—P1—Au1	113.6 (3)	C11—C12—C7	120.0
C14—P1—C1C	105.3 (6)	C11—C12—H12	120.0
C1D—P1—Au1	116.0 (12)	C4A—S1A—C1A	96.8 (12)
C7—P1—Au1	113.6 (5)	S1A—C1A—P1	116.5 (13)
C7—P1—C1D	106.9 (14)	C2A—C1A—P1	137.1 (15)
C7—P1—C1B	102.5 (8)	C2A—C1A—S1A	106.2 (15)
C1A—P1—Au1	106.8 (9)	C1A—C2A—H2A	122.7
C1B—P1—Au1	111.1 (8)	C1A—C2A—C3A	115 (2)
C1B—P1—C1D	105.5 (12)	C3A—C2A—H2A	122.7
C1—P1—Au1	111.9 (5)	C2A—C3A—H3A	123.4
C1—P1—C14	106.6 (6)	C4A—C3A—C2A	113 (2)
C1—P1—C1C	106.9 (11)	C4A—C3A—H3A	123.4
C1C—P1—Au1	112.1 (6)	S1A—C4A—H4A	125.5
C15—C14—P1	117.4 (4)	C3A—C4A—S1A	109.0 (19)
C15—C14—C19	120.0	C3A—C4A—H4A	125.5
C19—C14—P1	122.6 (4)	C4B—S1B—C1B	95.0 (9)
C14—C15—H15	120.0	S1B—C1B—P1	117.1 (9)
C16—C15—C14	120.0	C2B—C1B—P1	135.4 (10)
C16—C15—H15	120.0	C2B—C1B—S1B	107.5 (12)
C15—C16—H16	120.0	C1B—C2B—H2B	122.9
C15—C16—C17	120.0	C1B—C2B—C3B	114.2 (16)
C17—C16—H16	120.0	C3B—C2B—H2B	122.9
C16—C17—H17	120.0	C2B—C3B—H3B	124.0
C18—C17—C16	120.0	C4B—C3B—C2B	111.9 (18)
C18—C17—H17	120.0	C4B—C3B—H3B	124.0
C17—C18—H18	120.0	S1B—C4B—H4B	124.5
C17—C18—C19	120.0	C3B—C4B—S1B	110.9 (15)
C19—C18—H18	120.0	C3B—C4B—H4B	124.5
C14—C19—H19	120.0	C2—C1—P1	118.3 (6)
C18—C19—C14	120.0	C2—C1—C6	120.0
C18—C19—H19	120.0	C6—C1—P1	121.5 (6)
C4D—S1D—C1D	97.6 (11)	C1—C2—H2	120.0
S1D—C1D—P1	121.1 (11)	C1—C2—C3	120.0
C2D—C1D—P1	132.8 (13)	C3—C2—H2	120.0
C2D—C1D—S1D	106.1 (14)	C2—C3—H3	120.0
C1D—C2D—H2D	123.1	C2—C3—C4	120.0
C1D—C2D—C3D	113.7 (18)	C4—C3—H3	120.0
C3D—C2D—H2D	123.1	C3—C4—H4	120.0
C2D—C3D—H3D	122.8	C5—C4—C3	120.0
C4D—C3D—C2D	114 (2)	C5—C4—H4	120.0
C4D—C3D—H3D	122.8	C4—C5—H5	120.0
S1D—C4D—H4D	126.4	C4—C5—C6	120.0
C3D—C4D—S1D	107.3 (16)	C6—C5—H5	120.0
C3D—C4D—H4D	126.4	C1—C6—H6	120.0
C8—C7—P1	121.2 (6)	C5—C6—C1	120.0
C8—C7—C12	120.0	C5—C6—H6	120.0
C12—C7—P1	118.8 (6)	C4C—S1C—C1C	92.8 (8)
C7—C8—H8	120.0	S1C—C1C—P1	119.5 (8)
C7—C8—C9	120.0	C2C—C1C—P1	129.2 (7)
C9—C8—H8	120.0	C2C—C1C—S1C	111.0 (10)
C8—C9—H9	120.0	C1C—C2C—H2C	124.5
C10—C9—C8	120.0	C1C—C2C—C3C	111.0 (13)
C10—C9—H9	120.0	C3C—C2C—H2C	124.5
C9—C10—H10	120.0	C2C—C3C—H3C	123.4
C11—C10—C9	120.0	C4C—C3C—C2C	113.2 (14)
C11—C10—H10	120.0	C4C—C3C—H3C	123.4
C10—C11—H11	120.0	S1C—C4C—H4C	124.1
C10—C11—C12	120.0	C3C—C4C—S1C	111.8 (12)
C12—C11—H11	120.0	C3C—C4C—H4C	124.1
			
Au1—P1—C14—C15	−59.1 (5)	C7—P1—C1B—S1B	−167.2 (16)
Au1—P1—C14—C19	119.9 (4)	C7—P1—C1B—C2B	10 (3)
Au1—P1—C7—C8	−27.0 (10)	C7—C8—C9—C10	0.0
Au1—P1—C7—C12	153.9 (6)	C8—C7—C12—C11	0.0
Au1—P1—C1A—S1A	145.5 (19)	C8—C9—C10—C11	0.0
Au1—P1—C1A—C2A	−30 (4)	C9—C10—C11—C12	0.0
Au1—P1—C1B—S1B	−45 (2)	C10—C11—C12—C7	0.0
Au1—P1—C1B—C2B	132 (3)	C12—C7—C8—C9	0.0
Au1—P1—C1—C2	−39.9 (9)	S1A—C1A—C2A—C3A	4 (3)
Au1—P1—C1—C6	134.8 (6)	C1A—S1A—C4A—C3A	1 (2)
Au1—P1—C1C—S1C	152.2 (14)	C1A—C2A—C3A—C4A	−3 (3)
Au1—P1—C1C—C2C	−33 (3)	C2A—C3A—C4A—S1A	1 (3)
P1—C14—C15—C16	179.0 (7)	C4A—S1A—C1A—P1	−179 (3)
P1—C14—C19—C18	−179.0 (7)	C4A—S1A—C1A—C2A	−3 (2)
P1—C1D—C2D—C3D	−177 (4)	S1B—C1B—C2B—C3B	−7 (2)
P1—C7—C8—C9	−179.1 (13)	C1B—P1—C7—C8	93.0 (13)
P1—C7—C12—C11	179.1 (13)	C1B—P1—C7—C12	−86.1 (14)
P1—C1A—C2A—C3A	179 (4)	C1B—S1B—C4B—C3B	−3.7 (17)
P1—C1B—C2B—C3B	176 (3)	C1B—C2B—C3B—C4B	4 (3)
P1—C1—C2—C3	174.8 (11)	C2B—C3B—C4B—S1B	0 (2)
P1—C1—C6—C5	−174.6 (12)	C4B—S1B—C1B—P1	−176 (2)
P1—C1C—C2C—C3C	−174 (2)	C4B—S1B—C1B—C2B	5.9 (18)
C14—P1—C1—C2	84.9 (8)	C1—P1—C14—C15	177.2 (5)
C14—P1—C1—C6	−100.4 (8)	C1—P1—C14—C19	−3.8 (7)
C14—P1—C1C—S1C	28 (2)	C1—P1—C1C—S1C	−84.9 (19)
C14—P1—C1C—C2C	−158 (2)	C1—P1—C1C—C2C	89 (2)
C14—C15—C16—C17	0.0	C1—C2—C3—C4	0.0
C15—C14—C19—C18	0.0	C2—C1—C6—C5	0.0
C15—C16—C17—C18	0.0	C2—C3—C4—C5	0.0
C16—C17—C18—C19	0.0	C3—C4—C5—C6	0.0
C17—C18—C19—C14	0.0	C4—C5—C6—C1	0.0
C19—C14—C15—C16	0.0	C6—C1—C2—C3	0.0
S1D—C1D—C2D—C3D	4 (4)	S1C—C1C—C2C—C3C	0 (2)
C1D—P1—C7—C8	−156.3 (10)	C1C—P1—C14—C15	63.9 (11)
C1D—P1—C7—C12	24.6 (12)	C1C—P1—C14—C19	−117.1 (10)
C1D—P1—C1B—S1B	81 (2)	C1C—P1—C1—C2	−162.9 (7)
C1D—P1—C1B—C2B	−101 (3)	C1C—P1—C1—C6	11.8 (9)
C1D—S1D—C4D—C3D	−6 (3)	C1C—S1C—C4C—C3C	2.5 (19)
C1D—C2D—C3D—C4D	−9 (5)	C1C—C2C—C3C—C4C	1 (2)
C2D—C3D—C4D—S1D	10 (4)	C2C—C3C—C4C—S1C	−3 (2)
C4D—S1D—C1D—P1	−178 (3)	C4C—S1C—C1C—P1	173.6 (19)
C4D—S1D—C1D—C2D	1 (3)	C4C—S1C—C1C—C2C	−1.6 (19)

### Hydrogen-bond geometry (Å, º)

*Cg*1, *Cg*2, *Cg*3, and *Cg*4 are the centroids of the S1*C*/C1*C*–C4*C*, S1*D*/C1*D*–C4*D*, C7–C12, and C14–C19 rings, respectively.

**Table:**

*D*—H···*A*	*D*—H	H···*A*	*D*···*A*	*D*—H···*A*
C2*C*—H2*C*···*Cg*2^i^	0.95	2.80	141	4 (1)
C2*C*—H2*C*···*Cg*4^i^	0.95	2.79	139	4 (1)
C3*D*—H3*D*···*Cg*1^ii^	0.95	2.87	141	4 (1)
C3*D*—H3*D*···*Cg*3^ii^	0.95	2.85	141	4 (1)
C8—H8···*Cg*2^i^	0.95	2.80	131	4 (1)
C8—H8···*Cg*4^i^	0.95	2.77	130	4 (1)
C18—H18···*Cg*1^ii^	0.95	2.96	129	4 (1)
C18—H18···*Cg*3^ii^	0.95	2.95	128	4 (1)

Symmetry codes: (i) −*x*+1, *y*+1/2, −*z*+3/2; (ii) −*x*+3/2, −*y*+1, *z*−1/2.
